# Supplementary material for: Modelling Skylarks (Alauda arvensis) to Predict Impacts of Changes in Land Management and Policy: Development and Testing of an Agent-Based Model
Source: PLoS One. 2013 Jun 6;8(6):e65803. doi: 10.1371/journal.pone.0065803 (PMC3675089; doi:10.1371/journal.pone.0065803)
Supplement: Supporting Information S4 — The skylark ODdox as a zipped archive. (ZIP) [file pone.0065803.s004.zip › Skylark_ODdox/class_polygon_data_vector.html]

ALMaSS Skylark ODdox: PolygonDataVector Class Reference


ALMaSS Skylark ODdox
 2.0


- Main Page
- Related Pages
- Classes
- Files

- Class List
- Class Index
- Class Hierarchy
- Class Members

Public Member Functions |
Private Attributes

PolygonDataVector Class Reference

`#include <CIPELandscapeMaker.h>`

List of all members.

|  |  |
| --- | --- |
| Public Member Functions | |
| void | AddPolygon (EGP\_Data egpdata) |
| bool | CanGrow (int polyref, int x, int y) |
| bool | HaveSeen (int a\_poly) |
|  | PolygonDataVector () |
|  | ~PolygonDataVector () |

|  |  |
| --- | --- |
| Private Attributes | |
| vector< EdgeGrowingPoints \* > | m\_polylist |
| int | m\_polyrefs [25000] |

---

## Constructor & Destructor Documentation

|  |  |  |  |  |
| --- | --- | --- | --- | --- |
| PolygonDataVector::PolygonDataVector | ( |  | ) |  |

|  |  |  |  |  |
| --- | --- | --- | --- | --- |
| PolygonDataVector::~PolygonDataVector | ( |  | ) |  |

---

## Member Function Documentation

|  |  |  |  |  |  |
| --- | --- | --- | --- | --- | --- |
| void PolygonDataVector::AddPolygon | ( | EGP\_Data | *egpdata* | ) |  |

|  |  |  |  |
| --- | --- | --- | --- |
| bool PolygonDataVector::CanGrow | ( | int | *polyref*, |
|  |  | int | *x*, |
|  |  | int | *y* |
|  | ) |  |  |

inline

References m\_polylist, and m\_polyrefs.

{ return m\_polylist[m\_polyrefs[polyref]]->GetCanGrow(x,y); }

|  |  |  |  |  |  |
| --- | --- | --- | --- | --- | --- |
| bool PolygonDataVector::HaveSeen | ( | int | *a\_poly* | ) |  |

inline

---

## Member Data Documentation

vector<EdgeGrowingPoints\*> PolygonDataVector::m\_polylist

private

Referenced by CanGrow().

int PolygonDataVector::m\_polyrefs[25000]

private

Referenced by CanGrow().

---

The documentation for this class was generated from the following file:

- CIPELandscapeMaker.h


- PolygonDataVector
- Generated on Thu Jan 10 2013 13:15:37 for ALMaSS Skylark ODdox by
   1.8.1.1
